# Migraine Headaches after Major Surgery with General or Neuraxial Anesthesia: A Nationwide Propensity-Score Matched Study

**DOI:** 10.3390/ijerph19010362

**Published:** 2021-12-30

**Authors:** Chung-Yi Liao, Chun-Cheng Li, Hsin-Yi Liu, Jui-Tai Chen, Yih-Giun Cherng, Tzeng-Ji Chen, Ying-Xiu Dai, Hsiang-Ling Wu, Wan-Chi Liu, Ying-Hsuan Tai

**Affiliations:** 1 Department of Anesthesiology, Shuang Ho Hospital, Taipei Medical University, New Taipei City 23561, Taiwan; 19206@s.tmu.edu.tw (C.-Y.L.); 15193@s.tmu.edu.tw (C.-C.L.); 18384@s.tmu.edu.tw (H.-Y.L.); 19240@s.tmu.edu.tw (J.-T.C.); stainless@s.tmu.edu.tw (Y.-G.C.); 2 Department of Anesthesiology, School of Medicine, College of Medicine, Taipei Medical University, Taipei 11031, Taiwan; 3 Department of Family Medicine, Taipei Veterans General Hospital, Taipei 11217, Taiwan; tjchen@vghtpe.gov.tw; 4 School of Medicine, National Yang Ming Chiao Tung University, Taipei 11221, Taiwan; daiinxiu@gmail.com (Y.-X.D.); hlwu9@vghtpe.gov.tw (H.-L.W.); 5 Department of Dermatology, Taipei Veterans General Hospital, Taipei 11217, Taiwan; 6 Department of Anesthesiology, Taipei Veterans General Hospital, Taipei 11217, Taiwan

**Keywords:** headache, postoperative, risk factor, spinal anesthesia, stress

## Abstract

Migraine headaches can be provoked by surgical stress and vasoactive effects of anesthetics of general anesthesia in the perioperative period. However, it is unclear whether general anesthesia increases the migraine risk after major surgery. Incidence and risk factors of postoperative migraine are also largely unknown. We utilized reimbursement claims data of Taiwan’s National Health Insurance and performed propensity score matching analyses to compare the risk of postoperative migraine in patients without migraine initially who underwent general or neuraxial anesthesia. Multivariable logistic regressions were applied to calculate the adjusted odds ratio (aOR) and 95% confidence interval (CI) for migraine risk. A total of 68,131 matched pairs were analyzed. The overall incidence of migraine was 9.82 per 1000 person-years. General anesthesia was not associated with a greater risk of migraine compared with neuraxial anesthesia (aORs: 0.93, 95% CI: 0.80–1.09). This finding was consistent across subgroups of different migraine subtypes, uses of migraine medications, and varying postoperative periods. Influential factors for postoperative migraine were age (aOR: 0.99), sex (male vs. female, aOR: 0.50), pre-existing anxiety disorder (aOR: 2.43) or depressive disorder (aOR: 2.29), concurrent uses of systemic corticosteroids (aOR: 1.45), ephedrine (aOR: 1.45), and theophylline (aOR: 1.40), and number of emergency room visits before surgery. There was no difference in the risk of postoperative migraine between surgical patients undergoing general and neuraxial anesthesia. This study identified the risk factors for postoperative migraine headaches, which may provide an implication in facilitating early diagnoses and treatment.

## 1. Introduction

Migraine is a complex neurovascular disorder that affects an estimated 15% of people worldwide [1,2]. Despite the recent advances in diagnosis and treatment, migraine remains the second leading cause of disability globally and accounts for the financial costs of USD 23 billion and more than EUR 50 billion in the USA and Europe, respectively [1,2].

Surgical patients are predisposed to migraine headaches due to stress, mental tension, and bright lights in the perioperative period [3,4]. Postoperative migraine headaches can cause emotional distress, induce sleep disorders, and impair health-related quality of life in surgical patients [1,2]. In addition, studies showed that patients with migraine have increased risks of some perioperative complications, including postoperative nausea and vomiting, ischemic stroke, and rehospitalization [5,6]. A prospective cohort study showed that the diagnosis of migraine was significantly associated with increased long-term risks of major cardiovascular diseases and cardiovascular mortality in women [7]. More efforts are needed to implement and improve migraine diagnosis and care in the postoperative period.

One of the theories of migraine pathogenesis is stimuli from vasodilation in intracranial arteries (mainly the branches of the middle meningeal artery), which are innervated by trigeminal nerve, to nociceptors in arterioles [8,9]. In animal studies, volatile anesthetics were found to have a vasodilatory effect on meningeal arterioles [10,11], and this effect may serve as a trigger of migraine after general anesthesia. By contrast, some studies reported the use of Propofol as the treatment of refractory migraine [12,13]. The overall effect of general anesthesia on the precipitation of migraine is poorly understood so far.

Cases studies have proposed that general anesthesia and opioids may be related to the development of postoperative migraine headaches [14,15,16,17,18]. Although several risk factors were reported for postoperative headache, it remains unclear whether general anesthesia increases the risk of postoperative migraine compared with other forms of anesthesia [3,19,20,21]. In addition, risk factors for postoperative migraine episodes are also largely unknown in current literature. The diagnosis of perioperative migraine can be difficult and complicated to establish if clinicians are unable to identify high-risk patients [14,16,17].

Accordingly, we utilized Taiwan’s National Health Insurance (NHI) research database to conduct the nationwide population-based cohort study. There are two objectives in this study. First, we aimed to compare the risk of postoperative migraine headaches between patients undergoing general and neuraxial anesthesia. Second, we sought to determine the risk factors for postoperative migraine headaches. Based on the current evidence [10,11,14,15,16,17,18], we hypothesized that general anesthesia was associated with a higher risk of postoperative migraine headaches compared with neuraxial anesthesia.

## 2. Materials and Methods

### 2.1. Source of Data

The present study was approved by the Institutional Review Board of Taipei Medical University in Taiwan (TMU-JIRB-N202101005). Written informed consent was waived by the Institutional Review Board. All methods of this study were performed in accordance with the STROBE guidelines and regulations. Taiwan’s National Health Insurance program was launched in March 1995 and provided insurance to more than 99% of Taiwan’s 23.4 million residents at the end of 2013. The NHI research database contains comprehensive data of the insured individuals, including demographic attributes (date of birth, sex, and residential location) and claims data (outpatient and inpatient care, medical diagnoses, prescriptions, and procedures). To protect personal privacy, a unique identification number is assigned to each beneficiary and enciphered before the data are released for research purposes. The NHI research database has been widely used in numerous epidemiological studies [22,23,24,25]. The present study used three Longitudinal Health Insurance Databases (LHID2000, LHID2005, and LHID2010), which randomly sampled 1 million beneficiaries from the original NHI research database in the years 2000, 2005, and 2010, respectively. The LHIDs contain the most updated medical claims of sampled beneficiaries since 1997. The representativeness of LHIDs has been validated by Taiwan’s National Health Research Institutes [26].

### 2.2. Study Population and Exposure Factors

We used the medical claims of 3 million insured beneficiaries to select patients who underwent their first surgical procedures requiring general or neuraxial anesthesia with a length of hospital stay ≥2 days in Taiwan between 1 January 2002 and 30 June 2013. We excluded surgeries which could only be performed with general anesthesia, patients who had any diagnoses of migraine in the outpatient or inpatient care within 24 months before the index surgery, and those who had a diagnosis of headache related to dural puncture or died within 180 days after the index surgery. Each subject with general anesthesia was randomly matched to a subject with neuraxial anesthesia in a ratio 1:1, using a frequency matched pair procedure.

### 2.3. Outcome of Interest

We identified patients who developed postoperative migraine headaches within 180 days after the index surgery by using the International Classification of Diseases, 9th Revision, Clinical Modification (ICD-9-CM) codes. (Supplementary Table S1) For sensitivity tests, migraine episodes in the varying postoperative periods (30, 60, 90, 120, and 150 days) were also compared between groups. The diagnosis of migraine was made by board-certified neurologists. Uses of acute migraine medications within 180 days after surgery were also examined, including sumatriptan, rizatriptan, ergotamine, and dihydroergotamine [27].

### 2.4. Covariates

Surgical procedures were classified into orthopedic (lower limbs), genitourinary, anal, obstetric, and hernia repair surgeries. We used the ICD-9-CM codes of physicians’ diagnoses within 24 months prior to surgery to ascertain the history of the following coexisting diseases, chosen based on data availability, physiological plausibility, and the existing literature: hypertension, diabetes mellitus, ischemic heart disease, atherosclerosis, heart failure, cerebrovascular disease, chronic kidney disease, chronic obstruction pulmonary disease, malignancy, anxiety disorder, depressive disorder, schizophrenia, and bipolar disorder [28]. Lifestyle factors included obesity, smoking disorder, alcohol use disorder, and malnutrition [28]. (Supplementary Table S1) Since adverse events after surgery may produce physical and emotional stress and trigger migraine headaches [3,4], we also analyzed the major complications that occurred within 30 days after the index surgery, including pneumonia, septicemia, acute renal failure, pulmonary embolism, deep vein thrombosis, stroke, urinary tract infection, surgical site infection, acute myocardial infarction, cardiac dysrhythmias, and postoperative bleeding. Perioperative uses of blood transfusion [29,30,31] and need for intensive care [32] during the index surgical admission were analyzed. Our analyses also adjusted for the commonly used sympathomimetic drugs prescribed within 180 days after the index surgery, which might affect cerebral blood flow and modify migraine risk, including systemic corticosteroids, ephedrine, and theophylline [33,34,35].

### 2.5. Statistical Analysis

A non-parsimonious multivariable logistic regression model was applied to estimate a propensity score for subjects undergoing general or neuraxial anesthesia. Each subject with general anesthesia was matched to a subject with neuraxial anesthesia using a greedy matching algorithm within a tolerance limit of 0.05 and without replacement to adjust for age, sex, insurance premium, types of surgery, comorbidities, lifestyle factors, concurrent sympathomimetic drugs, number of hospitalizations, and number of emergency room visits within 24 months before the index surgery. Categorical variables were expressed using frequency and percentage, and continuous variables were summarized using mean and standard deviation. The distributions of baseline attributes in propensity-score matched samples were compared between groups by using standardized difference [36]. Multivariable logistic regressions models were used to calculate the adjusted odds ratio (aOR) and 95% confidence interval (CI) of postoperative migraine headaches. We considered a two-sided level of 0.05 statistically significant. All the statistical analyses were conducted using Statistics Analysis System (SAS), Version 9.4 (SAS Institute Inc., Cary, NC, USA).

## 3. Results

### 3.1. Baseline Patient Characteristics

**Figure 1:**
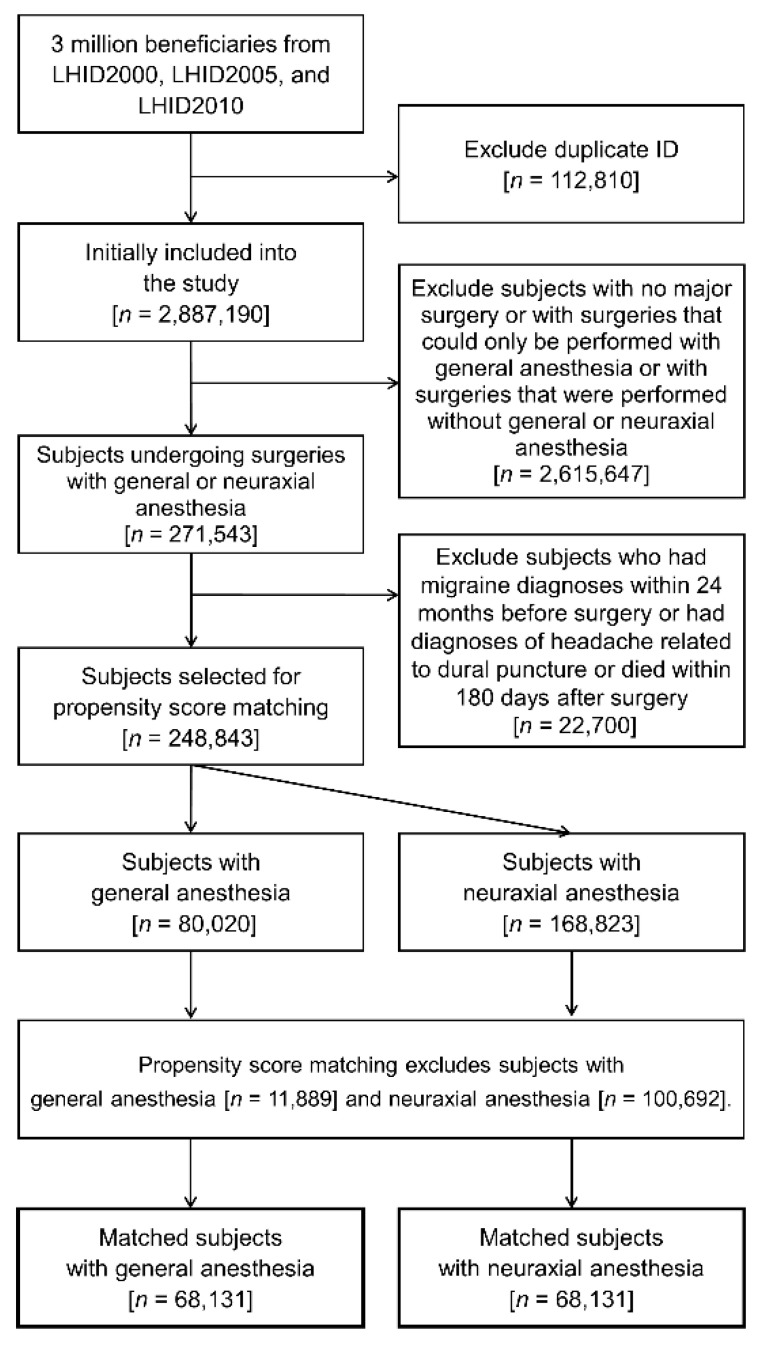
Flow diagram for patient selection.

After meeting the patient selection criteria, the matching procedure generated 68,131 matched pairs with 66,989 person-years of follow-up for analyses. (Figure 1) Table 1 shows the baseline characteristics of the included subjects undergoing general or neuraxial anesthesia. The distributions of demographics, types of surgery, comorbidities, lifestyle factors, concurrent sympathomimetic drugs, number of hospitalizations, and number of emergency room visits were well balanced after matching.

### 3.2. Risk of Postoperative Migraine Headaches

In the matched cohort, 658 patients developed a new-onset migraine during the half-year follow-up, and 318 and 340 after general and neuraxial anesthesia, respectively. The overall incidence of migraine was 9.82 per 1000 person-years, and 9.49 and 10.15 for patients undergoing general and neuraxial anesthesia, respectively.

Table 2 shows the results of univariate and multivariable logistic regression models for the risk of postoperative migraine headaches. There was no significant difference in the migraine risk between general and neuraxial anesthesia, aOR: 0.93 (95% CI: 0.80–1.09). Independent influential factors for migraine were age (aOR: 0.99), sex (male vs. female, aOR: 0.50), pre-existing anxiety disorder (aOR: 2.43), depressive disorder (aOR: 2.29), concurrent uses of systemic corticosteroids (aOR: 1.45), ephedrine (aOR: 1.45), and theophylline (aOR: 1.40), and number of emergency room visits before surgery (1 vs. 0, aOR: 1.12; 2 vs. 0, aOR: 1.14; ≥3 vs. 0, aOR: 1.68).

### 3.3. Subgroup Analyses

There was no significant difference in the migraine risk between patients undergoing general or neuraxial anesthesia across the subgroups of different migraine subtypes, uses of migraine medications, or varying postoperative periods. (Table 3) There was no difference between general and neuraxial anesthesia in migraine risk across the subgroups of age ≥ or <65 years, sex, anxiety disorder, depressive disorder, concurrent uses of systemic corticosteroids, ephedrine, theophylline, postoperative complications, or admission to intensive care unit, either (Table 4).

## 4. Discussion

In this study, we found that there was no significant difference in the risk of postoperative migraine headaches between patients undergoing general or neuraxial anesthesia. The results were consistent across subgroups of different migraine subtypes, uses of migraine medications, and varying postoperative periods. Importantly, our analyses identified several risk factors for postoperative migraine headaches, including younger age, female, pre-existing anxiety disorder, depressive disorder, and a greater number of preoperative emergency visits. The perioperative uses of systemic corticosteroid, ephedrine, and theophylline were associated with increased risks of migraine after surgery. To our knowledge, this is the first large-scale study to evaluate the overall incidence and potential risk factors for migraine headaches after major surgery. These findings provide evidence for the diagnosis, risk-stratification, and treatment of migraine headaches after major surgery.

Epidemiological study has reported that overall incidence of migraine was estimated at 8.1 per 1000 person-years in people without migraine initially [37]. In our study, the incidence of postoperative migraine headaches was 9.82 per 1000 person-years in the half-year follow-up, which was 21% higher than that of previous reports in general population [37]. Patients in our cohort were under the stress of surgery, which has been identified as the most important trigger of migraine [3,4,38]. Moreover, the present study showed that patients with pre-existing anxiety or depressive disorder had a greater risk of developing postoperative migraine. Emotional disturbances were found commonly comorbid with migraine in the general population [39,40]. The causation between anxiety/depressive disorders and migraine was unclear in the perioperative period. It is also uncertain whether the stress of surgery modifies the migraine risk among patients with mental health disorders. Our analyses revealed that more emergency room visits prior to surgery were associated with a higher risk of postoperative migraine. Two observational studies demonstrated a relationship between emergency room visits and severity of migraine [41,42]. In our study, preoperative frequency of emergency visits might reflect disease severity and surgical urgency, which potentially underlay the higher risk of postoperative migraine.

Our results refuted the primary hypothesis that general anesthesia was associated with a higher risk of postoperative migraine compared with neuraxial anesthesia. Until now, there are only few case studies reporting migraine headaches after general anesthesia and fentanyl sedation [14,15,16,17,18]. The association between general anesthesia and postoperative migraine episodes has been hypothesized, but there is still no study to compare the migraine risk between general anesthesia and other types of anesthesia. We raised the following possible explanations for our results. First, although the vasodilatory effect of volatile anesthetics on meningeal arterioles has been proposed to trigger migraine headaches [10,11], this neurovascular effect may be outweighed by Propofol used in general anesthesia. Propofol is an agonist of gamma-aminobutyric acid receptors, which inhibit the activity of central serotonergic neurons in the raphe nuclei and theoretically exert anti-migraine effects [12,13]. In addition, Propofol reduces both cerebral blood flow and cerebral metabolic rate, which may prevent the development of migraine [12,13]. Future studies are needed to investigate the potential impact of different regimens of general anesthesia on the risk of postoperative migraine, such as Propofol-based total intravenous anesthesia and opioid-free general anesthesia.

Our results suggested that uses of systemic corticosteroid, ephedrine, and theophylline were correlated with increased migraine risks. First, systemic corticosteroid has been used as an acute migraine treatment, and it is particularly effective for patients with refractory migraine, a history of recurrent headaches, and status migrainosus [43,44,45]. In our study, the use of systemic corticosteroid may represent a rescue therapy for migraine in these patients. Second, ephedrine is a non-selective beta agonist with alpha-1 activity [46,47]. In the current practice guideline, beta blockers without intrinsic sympathomimetic activity are used to prevent recurrent migraine headaches [46]. In a previous study conducted on healthy males, intravenous infusion of ephedrine caused an increase in blood flow of external carotid artery [47]. We reasoned that the increased blood flow of external carotid artery may increase the blood flow and induce vasodilation in the branches of middle meningeal artery, causing stimuli to trigeminal nerve and thereby triggering migraine [8,9]. Third, theophylline is a phosphodiesterase inhibiting agent used as the therapy for chronic obstructive pulmonary disease and asthma [48]. Theophylline has a narrow therapeutic window and its overdose can stimulate the central nervous system and cause headaches, insomnia, and seizure [49]. Besides, previous studies have shown that patients with asthma had a higher prevalence and incidence of migraine [50,51]. However, the mechanism is still unknown. Future studies are warranted to investigate the potential neurophysiological effects of theophylline on migraine headaches.

The present study discovered some potentially modifiable factors for migraine headaches after surgery, including systemic corticosteroids, ephedrine, and theophylline. Whether avoiding these drugs prevents the occurrence of migraine remains to be seen. It is also unclear what proportion of postoperative migraine progresses to chronic migraine. To date, the proven effective treatment for chronic migraine includes oral medications, neuromodulation, and nerve blockade [28,52]. Meta-analyses showed that acupuncture as a treatment modality was associated with a significant reduction in both headache frequency and response compared with routine care only and with prophylactic drugs at 2 months [53]. However, these results were potentially limited by the heterogeneity across studies and placebo effects [53]. Future studies are warranted to better evaluate the potential benefits and adverse effects of acupuncture in the prevention and treatment of chronic migraine.

There were some limitations to our study. First, our data did not contain information about physical measures (e.g., blood pressure), biochemical laboratory tests (e.g., stress hormone levels), socioeconomic factors (e.g., education level), and clinical data on detailed surgical (elective, urgent or emergent surgery, and duration of surgery) and anesthetic management (uses of vasoconstrictors or vasodilators, types and doses of anesthetics and opioids) that were not covered by NHI database. Second, our analyses have excluded patients who had a diagnosis of chronic migraine before surgery and therefore it is unclear whether types of anesthesia affect the precipitation of migraine headaches in this population. Third, surgeries that could only be performed with general anesthesia (e.g., brain surgery and ear, nose, and throat surgery) were excluded from analyses, which might decrease the generalizability of study results [32]. Fourth, a recent study reported that pre-existing migraine was a risk factor for postoperative nausea and vomiting [5]. However, primary care physicians rarely used diagnosis codes to document postoperative nausea and vomiting in the NHI research database probably due to its transient and mild symptoms compared with active surgical problems. This precluded meaningful analyses of the relationship between migraine headaches and postoperative nausea and vomiting in this study. Fifth, our dataset did not include patients with migraine who did not seek conventional medical care due to mild symptoms. Sixth, we did not evaluate the risk of migraine among people who did not undergo surgery or anesthesia. Finally, our cohort was only followed up until the end of 2013, due to the regulations of the NHI research database.

## 5. Conclusions

There was no significant difference in the incidence of postoperative migraine headaches in patients undergoing general or neuraxial anesthesia for surgeries that could be performed with both techniques. Several risk factors for migraine were determined, including younger age, female, pre-existing depressive and anxiety disorders, concurrent uses of systemic corticosteroids, ephedrine, and theophylline and number of emergency room visits before surgery. These findings may provide an implication for early diagnoses and prompt interventions of postoperative migraine headaches. More studies are needed to diagnose and evaluate postoperative migraines among patients receiving different anesthetics in the immediate postoperative period.

## Figures and Tables

**Table 1:** Baseline characteristics of patients undergoing general and neuraxial anesthesia.

Baseline Characteristic	General Anesthesia *n* = 68,131		Neuraxial Anesthesia *n* = 68,131		SDD
Age (years), mean (SD)	51.4	19.8	51.1	19.5	0.0153
Sex, male, *n* (%)	36,890	54.2	37,003	54.3	−0.0037
Insurance premium (USD/month), *n* (%)					0.0028
0–500	29,888	43.9	30,182	44.3	
501–800	22,810	33.5	22,375	32.8	
≥801	15,433	22.7	15,574	22.9	
Type of surgery, *n* (%)					
Orthopedic, lower limbs	29,824	43.8	29,629	43.5	0.0064
Genitourinary	18,018	26.5	17,692	26.0	0.0136
Anal	7373	10.8	7613	11.2	−0.0198
Obstetric	6972	10.2	6966	10.2	0.0005
Hernia repair	6168	9.1	6506	9.6	−0.0324
Lifestyle factors, *n* (%)					
Obesity	371	0.5	381	0.6	−0.0147
Smoking disorder	434	0.6	437	0.6	−0.0038
Alcohol use disorder	945	1.4	925	1.4	0.0120
Malnutrition	477	0.7	486	0.7	−0.0104
Comorbidity, *n* (%)					
Hypertension	20,416	30.0	20,149	29.6	0.0103
Diabetes mellitus	9719	14.3	9542	14.0	0.0118
Ischemic heart disease	7363	10.8	7357	10.8	0.0005
Atherosclerosis	604	0.9	594	0.9	0.0093
Heart failure	2242	3.3	2212	3.3	0.0077
Cerebrovascular disease	5453	8.0	5429	8.0	0.0026
Chronic kidney disease	3708	5.4	3692	5.4	0.0025
COPD	5663	8.3	5685	8.3	−0.0023
Malignancy	4478	6.6	4446	6.5	0.0042
Anxiety disorder	7127	10.5	7131	10.5	−0.0003
Depressive disorder	723	1.1	734	1.1	−0.0084
Schizophrenia	383	0.6	374	0.6	0.0132
Bipolar disorder	268	0.4	281	0.4	−0.0262
Concurrent sympathomimetic drugs, *n* (%)					
Systemic corticosteroids	11,439	16.8	11,291	16.6	0.0086
Ephedrine	11,961	17.6	12,099	17.8	−0.0077
Theophylline	6623	9.7	6702	9.8	−0.0072
Number of hospitalizations, *n* (%)					0.0238
0	52,007	76.3	52,997	77.8	
1	10,502	15.4	9750	14.3	
2	3217	4.7	2934	4.3	
≥3	2405	3.5	2450	3.6	
Number of ER visits, *n* (%)					−0.0004
0	39,554	58.1	39,772	58.4	
1	16,202	23.8	15,864	23.3	
2	6423	9.4	6421	9.4	
≥3	5952	8.7	6074	8.9	
Postoperative complications, *n* (%)	8533	12.5	9105	13.4	−0.0411
Blood transfusion, *n* (%)	986	1.5	815	1.2	0.1064
ICU admission, *n* (%)	430	0.6	374	0.6	0.0774

Abbreviation: COPD = chronic obstruction pulmonary disease; ER = emergency room; ICU = intensive care unit; SD = standard deviation; SDD = standardized difference; and USD = United States dollar.

**Table 2:** Univariate and multivariable analyses for the risk of postoperative migraine.

	Univariate			Multivariable		
	cOR	95% CI	*p*	aOR	95% CI	*p*
General vs. neuraxial anesthesia	0.94	0.80–1.09	0.3900	0.93	0.80–1.09	0.3578
Age (years)	1.00	1.00–1.00	0.8573	0.99	0.99–1.00	0.0211
Sex, male	0.51	0.44–0.60	<0.0001	0.50	0.42–0.60	<0.0001
Insurance premium (USD/month)			0.5394			0.7854
501–800 vs. 0–500	0.98	0.83–1.17	0.6495	0.97	0.81–1.16	0.9443
≥801 vs. 0–500	0.89	0.73–1.09	0.2815	0.93	0.75–1.15	0.5540
Type of surgery						
Orthopedic, lower limbs	0.88	0.75–1.03	0.1136	0.43	0.06–3.14	0.4079
Genitourinary	0.95	0.80–1.13	0.5671	0.52	0.07–3.70	0.5096
Anal	1.32	1.06–1.64	0.0143	0.62	0.09–4.49	0.6356
Obstetric	1.20	0.95–1.52	0.1318	0.39	0.05–2.88	0.3592
Hernia repair	0.89	0.67–1.17	0.4044	0.59	0.08–4.23	0.6000
Lifestyle factors						
Obesity	1.66	0.74–3.73	0.2164	1.30	0.58–2.93	0.5261
Smoking disorder	1.44	0.64–3.22	0.3778	1.50	0.66–3.37	0.3327
Alcohol use disorder	0.55	0.23–1.33	0.1823	0.51	0.21–1.24	0.1359
Malnutrition	1.08	0.45–2.60	0.8655	0.84	0.35–2.05	0.7078
Comorbidity						
Hypertension	1.11	0.95–1.31	0.1966	0.92	0.75–1.14	0.4612
Diabetes mellitus	1.12	0.90–1.38	0.3135	1.03	0.82–1.30	0.8100
Ischemic heart disease	1.38	1.11–1.72	0.0041	1.12	0.87–1.45	0.3697
Atherosclerosis	1.57	0.81–3.03	0.1820	1.26	0.65–2.47	0.4948
Heart failure	1.32	0.90–1.93	0.1538	1.02	0.67–1.53	0.9442
Cerebrovascular disease	1.31	1.02–1.69	0.0374	1.20	0.90–1.60	0.2110
Chronic kidney disease	1.37	1.02–1.84	0.0352	1.04	0.76–1.43	0.8015
COPD	1.38	1.08–1.76	0.0104	1.16	0.89–1.51	0.2772
Malignancy	0.92	0.67–1.27	0.6252	0.93	0.67–1.30	0.6651
Anxiety disorder	3.05	2.56–3.64	<0.0001	2.43	2.01–2.95	<0.0001
Depressive disorder	4.18	2.85–6.12	<0.0001	2.29	1.53–3.44	<0.0001
Schizophrenia	1.10	0.41–2.94	0.8563	0.86	0.31–2.33	0.7588
Bipolar disorder	1.13	0.36–3.53	0.8296	0.51	0.16–1.63	0.2555
Concurrent sympathomimetic drugs						
Systemic corticosteroids	1.69	1.42–2.02	<0.0001	1.45	1.21–1.74	<0.0001
Ephedrine	1.80	1.52–2.14	<0.0001	1.45	1.21–1.75	<0.0001
Theophylline	1.84	1.50–2.26	<0.0001	1.40	1.12–1.74	0.0036
Number of hospitalizations			0.1994			0.2011
1 vs. 0	1.24	1.01–1.52	0.2302	1.05	0.85–1.30	0.0668
2 vs. 0	1.16	0.82–1.65	0.7173	0.87	0.60–1.27	0.9935
≥3 vs. 0	1.02	0.67–1.56	0.6570	0.64	0.40–1.01	0.0676
Number of ER visits			<0.0001			0.0012
1 vs. 0	1.13	0.93–1.37	0.1220	1.12	0.92–1.36	0.3045
2 vs. 0	1.20	0.92–1.56	0.5957	1.14	0.87–1.50	0.5517
≥3 vs. 0	1.89	1.51–2.37	<0.0001	1.68	1.30–2.16	0.0004
Postoperative complications	1.03	0.82–1.29	0.8300	0.92	0.73–1.17	0.5028
Blood transfusion	0.69	0.31–1.54	0.3608	0.58	0.24–1.39	0.2217
ICU admission	1.29	0.54–3.12	0.5686	1.55	0.59–4.06	0.3725

Abbreviation: aOR = adjusted odds ratio; COPD = chronic obstruction pulmonary disease; cOR = crude odds ratio; ER = emergency room; ICU = intensive care unit; USD = United States dollar.

**Table 3:** Risk of postoperative migraine stratified by migraine subtypes, migraine medications, and varying postoperative periods.

	General Anesthesia			Neuraxial Anesthesia			Migraine Risk		
	Event	Rate (%)	Crude Incidence Rate/1000 PY	Event	Rate (%)	Crude Incidence Rate/1000 PY	Incidence Rate Ratio	aOR (95% CI) †	*p*
All migraine	318	0.47	9.49	340	0.50	10.15	0.94	0.93 (0.80–1.09)	0.3578
Migraine with aura	32	0.05	0.96	31	0.05	0.93	1.03	1.02 (0.62–1.68)	0.9295
Migraine without aura	58	0.09	1.73	78	0.11	2.33	0.74	0.73 (0.52–1.03)	0.0697
Migraine, unspecified	228	0.33	6.81	231	0.34	6.90	0.99	0.99 (0.82–1.19)	0.8857
Migraine with medications	102	0.15	3.05	83	0.12	2.48	1.23	1.23 (0.92–1.64)	0.1717
30-day migraine	60	0.09	10.73	58	0.09	10.37	1.03	1.03 (0.72–1.48)	0.8706
60-day migraine	115	0.17	10.28	112	0.16	10.02	1.03	1.02 (0.79–1.33)	0.8793
90-day migraine	174	0.26	10.38	161	0.24	9.60	1.08	1.08 (0.87–1.34)	0.4981
120-day migraine	218	0.32	9.76	220	0.32	9.84	0.99	0.99 (0.82–1.19)	0.8893
150-day migraine	276	0.41	9.88	282	0.41	10.10	0.98	0.97 (0.82–1.15)	0.7570

Abbreviation: aOR = adjusted odds ratio; CI = confidence interval; PY = person-years. † Adjusted for age (continuous), sex, insurance premium (categorical), types of surgery, comorbidities, lifestyle factors, concurrent sympathomimetic drugs, number of hospitalizations, number of emergency room visits, postoperative complications, perioperative uses of blood transfusion, and intensive care unit care.

**Table 4:** Subgroup analyses of postoperative migraine for patients undergoing general and neuraxial anesthesia.

Subgroup		*n*	Event	Rate (%)	aOR (95% CI) †	*p*
Age ≥ 65 years	GA	19,894	77	0.39	0.94 (0.69–1.29)	0.6985
	NA	20,074	83	0.41	reference	
Age < 65 years	GA	48,237	241	0.50	0.93 (0.78–1.11)	0.3972
	NA	48,057	257	0.53	reference	
Male	GA	36,890	112	0.30	0.84 (0.65–1.08)	0.1672
	NA	37,003	136	0.37	reference	
Female	GA	31,241	206	0.66	0.98 (0.81–1.20)	0.8629
	NA	31,128	204	0.66	reference	
Anxiety disorder	GA	7127	77	1.08	0.80 (0.59–1.08)	0.1410
	NA	7131	95	1.33	reference	
No anxiety disorder	GA	61,004	241	0.40	0.98 (0.82–1.18)	0.8551
	NA	61,000	245	0.40	reference	
Depressive disorder	GA	723	13	1.80	0.79 (0.34–1.83)	0.5818
	NA	734	15	2.04	reference	
No depressive disorder	GA	67,408	305	0.45	0.94 (0.80–1.10)	0.4096
	NA	67,397	325	0.48	reference	
Use of systemic corticosteroids	GA	11,439	82	0.72	0.98 (0.72–1.33)	0.8769
	NA	11,291	84	0.74	reference	
No use of systemic corticosteroids	GA	56,692	236	0.42	0.92 (0.77–1.09)	0.3280
	NA	56,840	256	0.45	reference	
Use of ephedrine	GA	11,961	84	0.70	0.86 (0.64–1.15)	0.3119
	NA	12,099	99	0.82	reference	
No use of ephedrine	GA	56,170	234	0.42	0.96 (0.80–1.15)	0.6469
	NA	56,032	241	0.43	reference	
Use of theophylline	GA	6623	48	0.72	0.78 (0.53–1.14)	0.1996
	NA	6702	61	0.91	reference	
No use of theophylline	GA	61,508	270	0.44	0.96 (0.81–1.14)	0.6500
	NA	61,429	279	0.45	reference	
Postoperative complications complications	GA	8533	41	0.48	0.94 (0.61–1.43)	0.7581
	NA	9105	46	0.51	reference	
No postoperative complications	GA	59,598	277	0.46	0.93 (0.79–1.10)	0.3777
	NA	59,026	294	0.50	reference	
Admission to ICU	GA	430	3	0.70	0.97 (0.08–12.33)	0.9825
	NA	374	2	0.53	reference	
No admission to ICU	GA	67,701	315	0.47	0.93 (0.79–1.08)	0.3357
	NA	67,757	338	0.50	reference	

Abbreviation: aOR = adjusted odds ratio; CI = confidence interval; GA = general anesthesia; ICU = intensive care unit; NA = neuraxial anesthesia. † Adjusted for age (continuous), sex, insurance premium (categorical), types of surgery, comorbidities, lifestyle factors, concurrent sympathomimetic drugs, number of hospitalizations, number of emergency room visits, postoperative complications, perioperative uses of blood transfusion, and intensive care unit care.

## Data Availability

The data presented in this study are available on request from the corresponding author.

## Supplementary Materials

The following are available online at https://www.mdpi.com/article/10.3390/ijerph19010362/s1, Table S1: ICD-9-CM codes of covariates and outcomes.
