# Supplementary material for: Migraine Headaches after Major Surgery with General or Neuraxial Anesthesia: A Nationwide Propensity-Score Matched Study
Source: Int J Environ Res Public Health. 2021 Dec 30;19(1):362. doi: 10.3390/ijerph19010362 (PMC8744620; doi:10.3390/ijerph19010362)
Supplement: Supplementary file 1 [file ijerph-19-00362-s001.zip › ijerph-1488877-supplementary.pdf]

Table S1. ICD-9-CM codes of covariates and outcomes

**Comorbidity**	
Hypertension	401-405
Diabetes mellitus	250
Ischemic heart disease	410-414
Atherosclerosis	440
Heart failure	428
Cerebrovascular disease	430-438
Chronic kidney disease	585
Chronic obstructive pulmonary disease	490, 491, 496
Malignancy	140-208, 230-234
Anxiety disorder	300
Depressive disorder	296.2, 296.3
Schizophrenia	295
Bipolar disorder	296, except 296.2x, 296.3x, 296.9x, 296.82
Headache related to dural puncture	349.0
**Lifestyle factor**	
Obesity	278
Smoking disorder	V15.82, 305.1
Alcohol use disorder	291.0, 291.1, 291.2, 291.3, 291.4, 291.5, 291.8, 291.81, 291.82, 291.89, 291.9, 303.00-303.03, 303.90-303.93, 305.00-305.03, 357.5, 425.5, 535.30, 535.31, 571.0, 571.1, 571.2, 571.3, E860.0
Malnutrition	262, 263.0, 263.1, 263.8, 263.9, 783.22, 783.21, 799.4, V85.0, 260, 261
**Postoperative complication**	
Pneumonia	480-486
Septicemia	038
Acute renal failure	584
Pulmonary embolism	415.1
Deep vein thrombosis	451.11, 451.19, 451.2, 451.81, 451.9, 453.40-453.42, 453.8, 453.9
Stroke	430-437
Urinary tract infection	599.0
Surgical site infection	682, 682.6, 682.9, 686.8, 686.9, 998.5, 998.51, 998.59
Acute myocardial infarction	410
Cardiac dysrhythmias	427
Postoperative bleeding	998.1
**Outcome**	
Migraine	346
Migraine with aura	346.0, 346.5, 346.6
Migraine without aura	346.1, 346.7
Migraine, unspecified	346.2, 346.3, 346.4, 346.8
